# A multi-parameter investigation of elite archery: distinguishing individual and shared performance strategies during the aiming and follow-through phases

**DOI:** 10.3389/fspor.2025.1650300

**Published:** 2025-09-19

**Authors:** Raphaël Jacquot, Khaireddine Ben Mansour, Kévin Bouillet, Jean-Philippe Jehl, Gérôme Gauchard

**Affiliations:** ^1^ UR 3450 DevAH, Université de Lorraine, Nancy, France; ^2^ CARE Grand Est, Université de Lorraine, Nancy, France; ^3^ Faculty of Sport Science, Université de Lorraine, Nancy, France; ^4^ Institut Jean Lamour, Université de Lorraine, Nancy, France

**Keywords:** archery, performance, neuromuscular activity, postural control, body configuration, temporal strategies, elite athletes

## Abstract

**Introduction:**

Archery accuracy relies heavily on the aiming and follow-through phases, during which factors such as muscle activation, postural control, and drawing arm movement play key roles. This study aims to assess whether these performance determinants in high-level archery are consistent across athletes or reflect individual-specific strategies.

**Methods:**

Eight top-level French archers (4 women, 4 men) shot eight rounds of nine arrows at a 70 m target, using their personal equipment in a competition-like outdoor setting. Data on neuromuscular activity, postural control, body segment configuration and temporal strategies were collected during the aiming and follow-through phases. Arrow scores were grouped into high (10), mid (9), and low (≤8) for analysis.

**Results:**

At the group level, several muscles and postural control parameters were associated with performance during the aiming phase (seven muscles, three postural parameters, and mechanical clicker reaction time) and the follow-through phase (four muscles and three postural parameters). At the individual level, two parameters during aiming (medial deltoid activity on the bow side and aiming duration) and two parameters during the follow-through phase (upper trapezius activity on the drawing side and surface of center of pressure displacement) were identified as individual strategies.

**Discussion:**

These findings highlight both shared performance determinants and individualized strategies among elite archers, emphasizing that while technical approaches vary, certain biomechanical patterns remain crucial for optimal performance.

## 1 Introduction

Archery is an Olympic sport practiced in an outdoor environment, where athletes shoot arrows at a target 70 meters away. The objective is to score as many points as possible by aiming at a 122 cm diameter target. This target is comprised of 10 concentric scoring rings, each with a width of 61 mm, assigned point values ranging from 1 to 10. The centermost ring yields the highest score, and this standardized scoring methodology provides a quantitative basis for performance evaluation in the sport.

The archery shooting sequence is usually divided into several phases, as described in previous studies (1–3). Based on these works, Callaway, Wiedlack, and Heller (4) proposed a more detailed model to date, identifying six distinct phases: pre-shot, set-up, draw, aiming, clicker-release time, and follow-through. Among these, the aiming and follow-through are considered important to performance, as they are among the most frequently studied in the literature (5). The aiming phase, beginning from the touch of bowstring on archer's face to the start of move away from the fingers (4), is detailed for accuracy, as it encompasses the final postural stabilization and system alignment prior to arrow launch (6–8). Subsequently, the follow-through phase, corresponding to the phase where the string release until the archer first moves or arm downward (4), is indicative of execution quality, as it provides insight into the athlete's ability to maintain control, balance and consistency in the post-release period.

The scientific literature provides a comprehensive analysis of archery performance through its constituent phases (2, 5, 9–14). These investigations have identified a multifactorial set of performance determinants, including temporal strategy, neuromuscular activity, postural stability, bow sway, mechanical clicker reaction time (MCRT), upper limb configuration and tremors, heart rate and cardiopulmonary parameters, and hand kinematics.

Before the aiming phase, a coordinated action of both upper limbs is required to align the bow-arrow-target system. The drawing arm serves the dual function of orienting the arrow's trajectory and storing potential energy in the bow for propulsion. Consequently, the configuration of the upper body throughout the archery motion has been studied, with a particular focus on shoulder abduction and elbow flexion on the drawing side. During the aiming phase, arm tremors were reduced when shoulder abduction was 90°, compared to 100° or 110° (15). Similar results have been reported in studies comparing different skill levels. Shinohara Urabe (7) reported that elite archers exhibited lower shoulder abduction angles (99.0° ± 4.5°) during aiming compared to novice archers (107.2° ± 4.0°), and also showed reduced elbow flexion angles (138.3° ± 5.0°) during aiming compared to novice archers (143.6° ± 3.7°), suggesting that a less abducted shoulder and a less flexed elbow are characteristic of high-level performance.

Keeping an optimal upper limb configuration depends on using multiple muscles to generate and stabilize movement. Accordingly, scientific literature has characterized the activity of key upper body and core muscles during the aiming and follow-through phases (8, 11, 14, 16–20). For both phases, muscular activation patterns diverged based on expertise, revealing a distal-dominant recruitment in novices, contrasted with a proximal-dominant strategy in elite archers (12, 13). These variations emphasize the key role of muscle recruitment in optimizing performance, suggesting that effective muscle stabilization and activation are essential for achieving accuracy and consistency in shooting.

Research in other precision sports such as biathlon and rifle shooting has shown that postural control is a key factor in archery performance (21–24). During the aiming phase, smaller amplitude and smaller velocity of center of pressure (CoP) displacement along the shooting axis is correlated with higher arrow scores (6, 14). Postural sway during the follow-through phase continues from that in the aiming phase and is integral to managing the forces generated during shot execution (17). Furthermore, post-release postural dynamics influence performance, as a higher maximum velocity of the CoP has been associated with lower shooting outcomes (25).

The temporal dynamics of the aiming phase are key factors influencing the kinematics of the upper limbs, muscular activity, and postural control. Several investigations have explored the relationship between aiming duration and performance level, with shorter aiming durations generally associated with higher performance (6, 8, 14). However, Callaway, Wiedlack, and Heller (4) reported contrasting findings, showing that longer aiming durations could be linked to better outcomes. This discrepancy suggests that the optimal temporal strategy during the aiming phase remains unclear and may be subject to inter-individual differences, and needs further investigation.

The aiming phase ends when the clicker is released, which happens when the archer has transferred the bow's energy to the string. The clicker serves as a crucial auditory cue, indicating that the archer has achieved a consistent, full draw length and that the release sequence should be initiated. The interval between this auditory signal and the subsequent release of the arrow, defined as the mechanical clicker reaction time (MCRT), has been shown to correlate with performance. Shorter MCRT is associated with better performance, both between skill levels (11) and within elite archers (25).

The literature has identified key factors contributing to successful archery performance. However, due to individual differences in technique, each archer employs their own neuromuscular and temporal strategies (5). Indeed, even among archers of a comparable performance level, variability in muscle activation patterns and aiming duration can be observed. Furthermore, to resolve the discrepancies prevalent in the current literature, comprehensive and standardized evaluations of performance determinants within a single population are crucial to ensure the reliability and reproducibility of the findings.

To date, most studies have focused on isolated variables such as muscle activity, postural control, or aiming time (4, 6, 12, 17, 24, 26). These studies, while informative, generally overlook the complex interactions and potential interaction effects that exist between different performance determinants. Only one study (14) has simultaneously analyzed several factors, including muscle activity, postural control, aiming time and MCRT. However, this study was constrained by its small sample size, involving only four archers who each shot thirty arrows. This methodological gap necessitates future research designed to assess multiple performance determinants simultaneously across a larger cohort of participants and an increased volume of shots.

Most archery research has focused on inter-group comparisons to identify performance trends (8, 11–13, 16–18, 20). While this approach reveals general patterns, it often overlooks inter-individual variability in motor strategies. Consequently, it remains unclear whether the key determinants of elite performance are uniform across athletes, or whether individual archers adopt unique compensatory or performance-enhancing strategies.

To address this gap, this study employs a dual-level analysis. First, a group-level analysis will be conducted to identify common success factors across all participants. Second, an individual-level analysis will examine the unique combination and expression of these factors within each archer. This two-tiered methodology is critical for differentiating between fundamental principles of high performance and the personalized strategies. Therefore, the aim of this study is to determine whether performance determinants in high-level archery (postural control, neuromuscular activity, body configuration and temporal strategies) are consistent across a group of eight archers or specific to each archer.

## 2 Materials and methods

### 2.1 Participants

Eight healthy, voluntary, high-level French archers were recruited (4 women and 4 men; 19.1 ± 2.7 years old). In 2024, all were ranked in the top 20 of the French scratch classic archery 70-meter ranking. They compete on the French national circuit, with a mean best score was 654.62 ± 14.25 points out of 720. Their training volume is around 21 hours per week. None of the participants had sustained any injuries in the six months prior to the study.

All participants were informed about the measurements, purpose, and potential risks associated with the experimental setup and provided their written consent before data collection. The study was conducted in accordance with the guidelines of the Declaration of Helsinki and was approved by the Ethics Committee for Research in Science and Techniques of Physical and Sports Activities (CER STAPS, n°IRB00012476-2024-20-11-356).

### 2.2 Experimental protocol

After completing a 15-minute warm-up following their usual routine, each archer was equipped with a set of sensors. Participants performed eight rounds of nine arrows, maintaining their habitual shooting pace. The interval between rounds corresponded to the time required to retrieve arrows from the target. Throughout the protocol, participants used their own bows and arrows.

The experiment was conducted on an outdoor archery field. Archers stood inside a cabin with open windows, while targets were placed 70-meter away from the participant, corresponding to the standard distance used in Olympic and international competitions. The target face used was the official 122 cm FITA 70-meter target. Each arrow shot was categorized based on score: 10 (target center) was classified as “High”, 9 (first circle) as “Mid”, and 8 or below (other circles) as “Low”.

### 2.3 Instruments and data analysis

#### 2.3.1 Upper body and trunk neuromuscular activity

**Figure 1:**
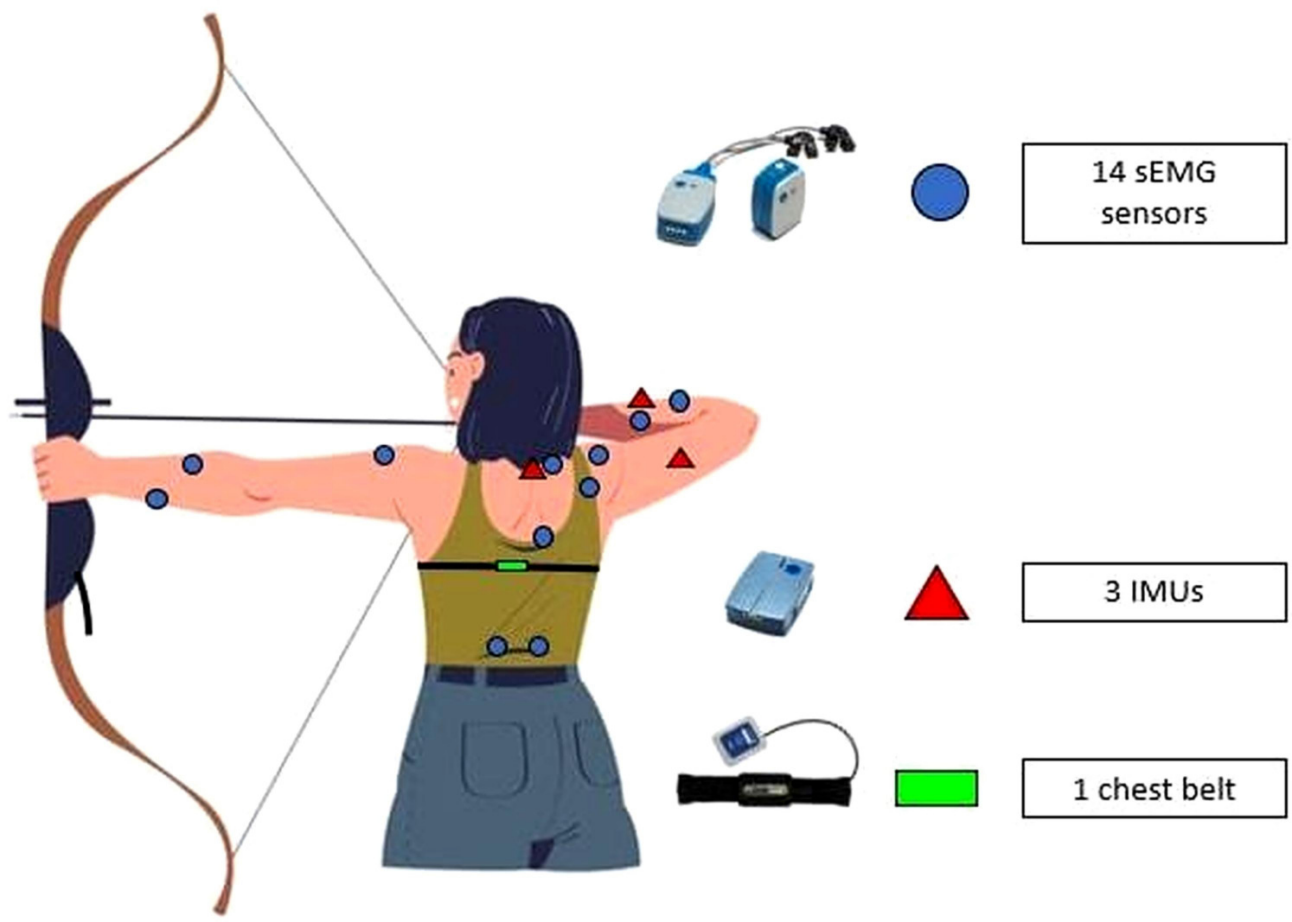
Sensors placement on the participant.

A wireless surface EMG system (Ultium EMG, Noraxon USA., Inc., Scottsdale, AZ, USA) with a sampling rate of 2,000 Hz was used to measure the neuromuscular activity from 14 trunk and upper limb muscles (Figure 1). A methodological precedent for using this instrument to assess these variables was established by Darendeli et al. (27). The choice of 14 muscles relies on their critical role in archery performance, as demonstrated in previous studies (8, 11, 14, 16–20). Before measurements, the skin was shaved, sanded, and cleaned to ensure low impedance, with a threshold below 5 kΩ considered acceptable for signal quality. Surface electrodes (Ambu® Blue-Sensor SP, Ambu A/S, Ballerup, Denmark) were placed parallel to the muscle fibers. A single experienced researcher placed all electrodes to maintain inter-rater consistency.

On the drawing arm side, electrodes were positioned over the extensor digitorum (ED-d), flexor digitorum superficialis (FDS-d), middle and posterior deltoid (respectively MD-d and PD-d), as well as the middle and upper trapezius (respectively MT-d and UT-d). On the bow arm side, flexor digitorum superficialis (FDS-b) and extensor digitorum (ED-b) were recorded, along with the middle deltoid (MD-b). Additionally, five core muscles were measured: the transvs. abdominis (TA), multifidus on both sides (MF-d and MF-b), and external oblique on both sides (EO-d and EO-b). Electrodes placement followed established guidelines (20, 28). For ED-d, ED-b, FDS-d, FDS-b, electrodes sites were identified by palpating the muscles while the subjects simulated the preparatory phase of the shooting position (11).

Before the first shooting round, maximum voluntary isometric contraction (MVIC) measurements were obtained for each muscle against static resistance, following the protocol described by Rota et al. (29) and Matsunaga et al. (20). The following protocols were used. ED: Forearm resting on a table and hand in a neutral position, maximum extension of the four fingers against resistance applied to the back of the fingers; FDS: maximum grip of a rowing oar handle with a diameter of 4 cm; MD: Arm lateral elevation at 90° abduction in the frontal plane with forearm pronated; PD: Horizontal abduction in the sagittal plane at 90° shoulder abduction with forearm pronated; UT: Standing, arm alongside the body, maximum shoulder elevation (shrugging) against downward resistance applied to the acromion; MT: In a lying position, with the arm extended in abduction at 90° and the thumb pointing upward, perform maximum scapular retraction against downward resistance; EO: Lying on back with knees bent and feet flat on the floor, perform a trunk flexion movement combined with a rotation, lifting one shoulder toward the opposite knee against manual resistance; TA: Standing up, perform a maximum abdominal contraction or “retraction” by pulling navel toward spine without moving pelvis or rib cage; ES: While lying down, perform a maximum extension of the trunk (by lifting the chest) with manual resistance applied to the upper back. EMG signals were bandpass filtered using a zero lag 4th-order Butterworth filter with a cut-off frequency of 20–500 Hz, then rectified and normalized to the corresponding MVIC values. For each muscle and each arrow, EMG data were averaged across the duration of both phases.

#### 2.3.2 Postural control

Center of pressure related parameters were recorded using an AccuPower force plate (AccuPower-O; AMTI, Watertown, MA, USA) at a sampling frequency of 1,000 Hz. The methodological choice of this instrument is supported by prior research (30), in which it was successfully utilized to assess the same biomechanical variables. The *Y* axis (called mediolateral) was aligned with the shooting direction, while the *X* axis (called anteroposterior) was perpendicular to it. Archers had to keep their feet still while shooting. The raw CoP coordinates were filtered using a 4th-order Butterworth low-pass filter with a 10 Hz cut-off frequency.

For each analyzed phase, the following variables were computed: the velocity of CoP displacement along the anteroposterior axis (Vx) and mediolateral axis (Vy), the resultant CoP velocity (V), the range of CoP displacement along the anteroposterior axis (Rx) and mediolateral axis (Ry), and the surface of CoP displacement (S). In addition, the maximum of CoP velocity (Vmax) was also calculated for each phase.

#### 2.3.3 Segmental alignment of the drawing arm

An IMU system (Ultium Motion, Noraxon USA., Inc., Scottsdale, AZ, USA) with a sampling rate of 200 Hz was used to measure the joint angles of the drawing arm. Sensors were positioned on the forearm, between the ulna and radius, on the lateral side of the arm, and at the C7 vertebra (Figure 1). The Noraxon IMU system has been validated for assessing shoulder kinematics (31). IMUs data were filtered using a 10 Hz, 4th-order low-pass filter. The mean values of shoulder abduction and elbow flexion on the drawing arm were calculated for both phases.

Kinematic and EMG signals were synchronized using the Noraxon system (Ultium Portable Lab, Noraxon USA, Inc., Scottsdale, AZ, USA).

#### 2.3.4 Chest movement

A chest belt (T-Sens Respiration, Technology Ergonomics Applications, Vandoeuvre-lès-Nancy, France) with a sampling rate of 32 Hz was placed at the level of the participant's tenth vertebra, as indicated by Decker et al. (32) (Figure 1). The range of chest movement (difference between maximal and minimal values during the phase) was calculated during both phases.

#### 2.3.5 Temporal strategies

Two cameras (GoPro Hero 9 Black, GoPro, San Mateo, CA, USA) with a 240 Hz sampling rate and 1080p resolution were used in this protocol. One was positioned to provide a wide field of view, capturing the entire participant to identify the aiming and follow-through phases and determine the duration of the aiming phase. This identification enabled the calculation of different variables independently for both phases of the archery movement. The second camera was focused on the participant's bow clicker to measure the MCRT using video analysis and the free 2D motion analysis software Kinovea (version 2023.1.2).

### 2.4 Statistics

All data processing steps, including filtering, rectification, normalization, and calculations, were performed using MATLAB software (R2022b, The MathWorks Inc., Natick, USA).

The analysis was structured in two phases. In the first phase, different parameters were compared according to score level groups (≤8, 9, 10) to identify performance determinants consistent at the group level. In the second phase, parameters that did not show significant group-level differences were analysed on an individual basis to identify subject-specific strategies. Thus, for each subject, parameter values across the different score groups (≤8, 9, 10) were compared.

For both analysis, Kruskal–Wallis tests were performed at both the group and the individual level. We selected this two-step non-parametric approach because it represents the most suitable method for our objective, given our small sample size (*N* = 8). When significant differences were found, *post-hoc* pairwise comparisons were conducted using Wilcoxon ranksum tests, with *p*-values adjusted using the Bonferroni correction. The statistical significance level was set at *p* < 0.05. All statistical analyses were performed using RStudio version 2024.12.1 (R Core Team, Vienna, Austria). Analyses were applied to the measured parameter (Table 1), including neuromuscular activity (14 parameters), body configuration (3 parameters), temporal strategies (2 parameters), and postural control (7 parameters).

**Table 1:** List of measured variables.

Measurement field	Variables (unit)
Muscular contribution	Percentage of neuromuscular activity relative to MVIC (%)
Postural control	Surface of 95% of CoP displacement (mm²)
	Range of CoP displacement in *X* axis and *Y* axis (mm)
	Mean velocity of CoP displacement (global, *X* axis, *Y* axis) and maximum velocity of global CoP displacement (mm/s)
Body configuration	Mean abduction of drawing shoulder (deg)
	Mean flexion of drawing elbow (deg)
	Range of movement of chest (a.u)
Temporal strategies	Mechanical clicker reaction time (ms)
	Total duration of aiming phase (s)

## 3 Results

To enhance clarity, only parameters showing significant differences will be illustrated in this section.

### 3.1 Scores

For each archer, the score corresponding to every arrow shot was systematically recorded. Due to measurement issues associated with the EMG sensors, IMU units, or force plate, some trials were excluded from the analysis. This led to variations in the number of arrows analysed per archer. The number of arrows scoring 8 or below (low), 9 (mid), and 10 (high) is presented in Table 2, for each participant, and for the entire group.

**Table 2:** Number of arrows for each score category for each archer.

Archers	Low	Mid	High	Total
A1	20	28	15	63
A2	12	29	31	72
A3	12	32	28	72
A4	18	22	32	72
A5	13	31	28	72
A6	23	32	17	72
A7	36	29	7	72
A8	29	17	8	54
Group	163	220	166	549

A1 to A8 represent archer 1 to archer 8.

### 3.2 Group comparisons

#### 3.2.1 Neuromuscular activity

**Figure 2:**
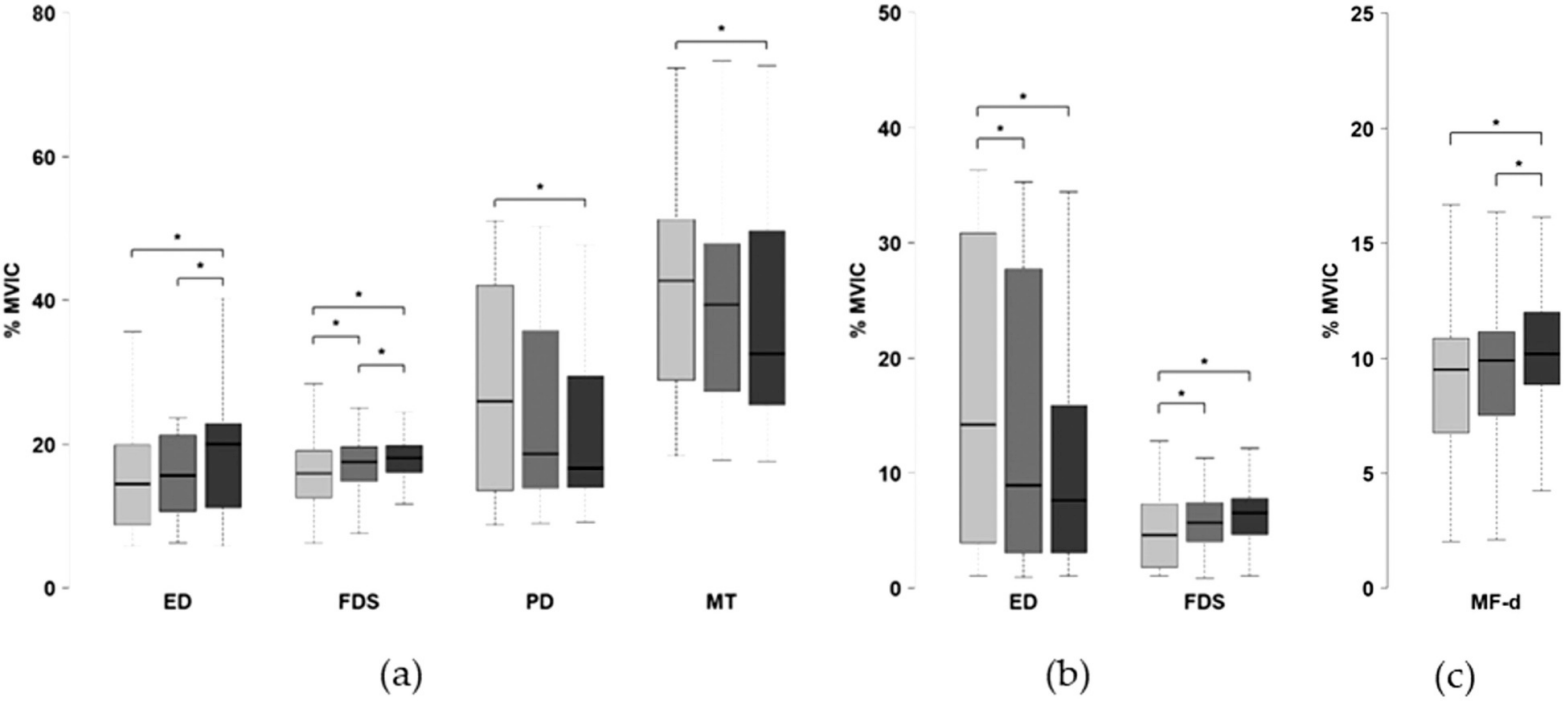
Neuromuscular activity value across score level (left: low; center: mid; right: high) during the aiming phase: **(a)** drawing arm muscles (ED, extensor digitorum; FDS, flexor digitorum superficialis; PD, posterior deltoid; MT, middle trapezius), of the **(b)** bow arm muscles (ED, extensor digitorum; FDS, flexor digitorum superficialis), of **(c)** core muscles (MF-d = multifidus drawing side). *p < 0.05.

At the group level, several parameters exhibited significant differences across scores during the aiming phase. Results related to the drawing arm, bow arm, and trunk muscles are presented in Figures 2a–c respectively. Neuromuscular activation of the posterior deltoid and middle trapezius on the drawing side was significantly higher during low scores compared to high scores, with no significant difference observed between low and mid scores. On the bow side, activation of the extensor digitorum was also significantly greater during low scores compared to mid and high scores.

Conversely, the extensor digitorum superficialis on the drawing side showed reduced activation during low scores compared to high scores, as well as during mid scores compared to high scores. Similarly, the flexor digitorum superficialis on the drawing side exhibited significantly lower activation for low scores compared to both mid and high scores, and for mid scores compared to high scores. On the bow side, the flexor digitorum superficialis showed lower activation during low scores compared to both mid and high scores. The multifidus muscle on the drawing side demonstrated reduced activation during low scores relative to high scores and also during mid scores compared to high scores. No significant differences were observed for the remaining muscles measured in this study.

**Figure 3:**
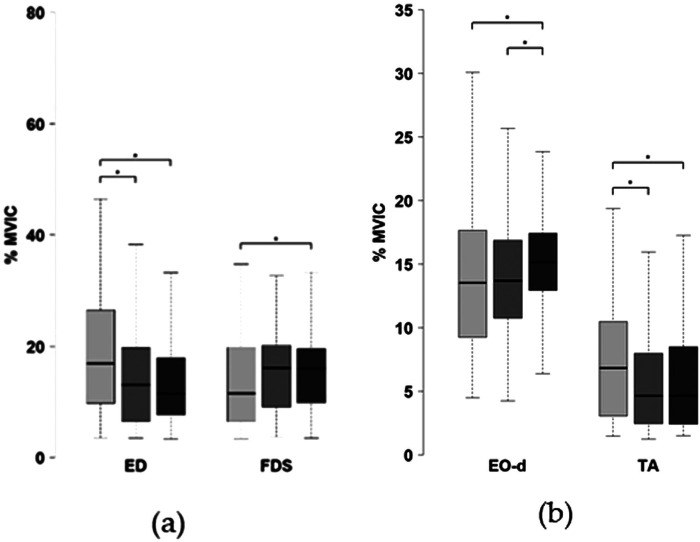
Neuromuscular activity value across score levels (left: low; center: mid; right: high) during the follow-through phase: **(a)** bow arm muscles (ED, extensor digitorum; FDS, flexor digitorum superficialis), and **(b)** core muscles (EO-d, external oblique drawing side; TA, transversus abdominis). * *p* < 0.05.

During the follow-through phase, neuromuscular activation of the extensor digitorum on the bow side was significantly greater during low scores compared to both mid and high scores (Figure 3a). Similarly, the transverse muscles showed higher activation during low scores than during mid and high scores (Figure 3b). Conversely, activation of the flexor digitorum superficialis on the bow side was significantly lower during low scores than during high scores, with no significant difference between low and mid scores (Figure 3a). Additionally, the external oblique on the drawing side was less activated during low scores compared to high scores and during mid scores compared to high scores (Figure 3b). No other significant differences in neuromuscular activation were observed for the remaining muscles.

#### 3.2.2 Postural control

**Figure 4:**
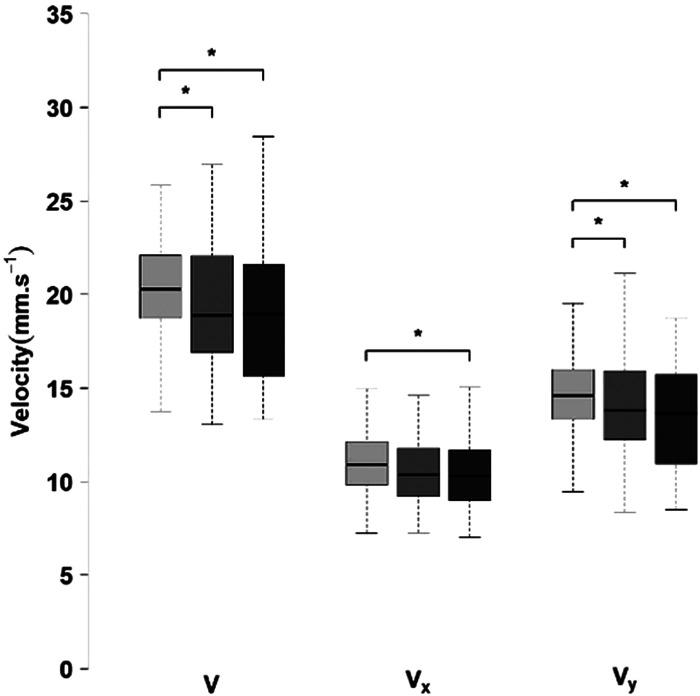
Postural control variables across score levels (left: low; center: mid; right: high) during the aiming phase (V, COP velocity; Vx, and Vy, COP velocity along the anteroposterior and mediolateral axis). *p < 0.05.

As shown in Figure 4, postural control during aiming phase was characterized by significant variations in CoP across scores levels. Specifically, the mean CoP velocity and mean CoP velocity along the mediolateral axis (Vy) were higher for low scores compared to both mid and high scores. In contrast, the mean CoP velocity along the anteroposterior axis (Vx) was significantly higher for low scores only when compared to high scores.

**Figure 5:**
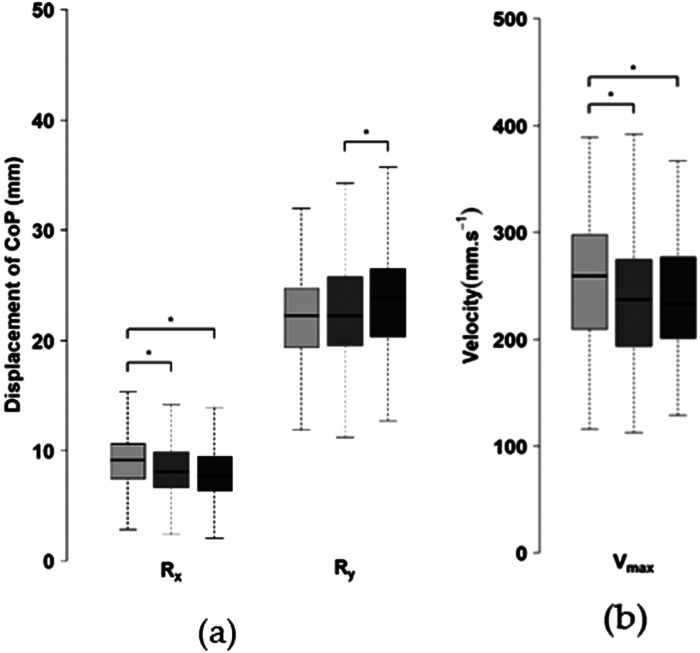
Postural control variables across score levels (left: low; center: mid; right: high) during the follow-through phase: **(a)** COP displacement along the anteroposterior (Rx) and mediolateral (Ry) axes; **(b)** maximum CoP velocity. *p < 0.05.

During the follow-through phase, the mediolateral displacement range of the CoP (Ry) was significantly smaller for mid-scores compared to high scores, with no significant difference with low scores (Figure 5a). Along the anteroposterior axis (Rx), CoP displacement was higher during low scores than during mid-range and high scores (Figure 5a). Furthermore, the maximum velocity of CoP displacement was greater during low scores than during mid and high scores (Figure 5b). No other significant differences were observed for the remaining postural control parameters.

#### 3.2.3 Body configuration

No significant differences in kinematic variables were found across score levels during either the aiming or follow-through phases.

#### 3.2.4 Temporal strategies

**Figure 6:**
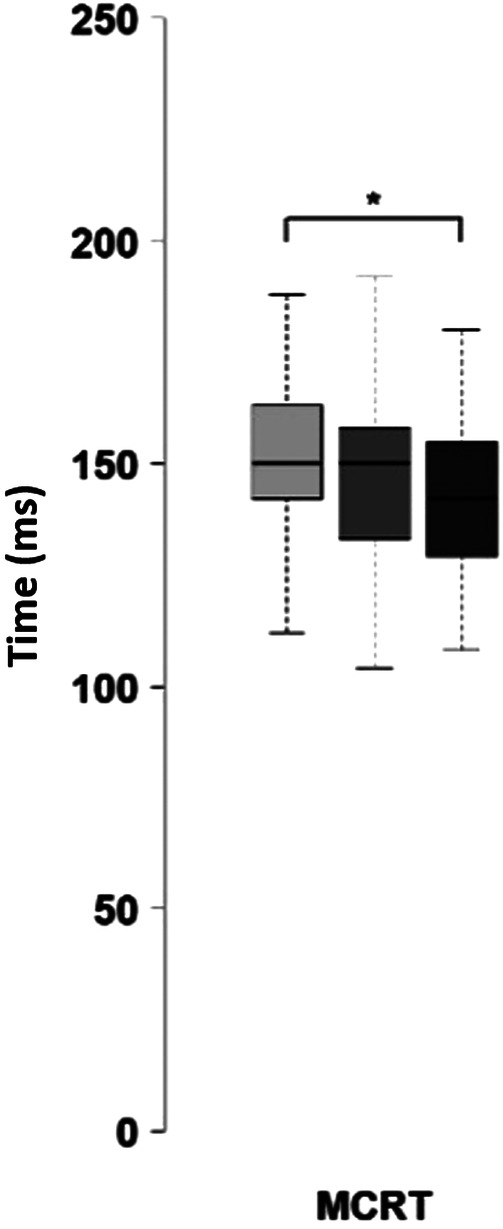
Temporal variables (MCRT, mechanical clicker reaction time) across different performance score levels (left: low; center: mid; right: high) during the aiming phase. * *p* < 0.05.

As shown in Figure 6, MCRT was significantly longer for low scores compared to high scores, with no difference with mid scores. No other significant differences were observed for the remaining variables.

#### 3.2.5 Individual comparisons

Among the 26 parameters analyzed, 11 related to aiming and 7 related to follow-through did not exhibit significant differences across score levels at the group level. This section explore which individual strategies archers may adopt to achieve their best performance.

#### 3.2.6 Neuromuscular activity

During the aiming phase, for A3, the middle deltoid on the drawing side was less active during high scores compared to mid scores. For the same archer, the posterior deltoid on the drawing side tended to be less active during high scores than during low (*p* = 0.058) and mid scores. Similarly, for A1, the external oblique on the drawing side showed reduced activation during high scores compared to low scores, with no significant difference between low and mid-scores. On the bow side, the middle deltoid displayed reduced activity during high scores compared to low scores for A1 and A8, and compared to mid-scores for A3. No other significant differences in muscle activation were observed among the remaining muscles or participants during the aiming phase. Muscle activation values for muscles showing significant differences per an archer during aiming phase are presented in Table 3.

**Table 3:** Neuromuscular activity values [median (IQR)], across scores level for each archer (FDS, flexor digitorum superficialis; MD, middle deltoid; PD, posterior deltoid; UT, upper trapezius; MT, middle trapezius; MF, multifidus; EO, external oblique; -d, drawing side; -b, bow side).

Phase	Variable	Archer	Low	Mid	High	*p*-value	*ε*²
Aiming	MD-d (%)	3	33.9 (2.7)	35.1 (4.1)^c^	31.8 (4.9)^b^	0.005	0.12
	MD-b (%)	1	59.9 (4.0)^c^	58.5 (4.5)	58.0 (3.4)^a^	0.031	0.08
	MD-b (%)	3	48.4 (6.0)	50.3 (6.3)^c^	46.5 (7.1)^b^	0.004	0.13
	MD-b (%)	8	65.5 (3.4)^c^	63.9 (3.2)	58.8 (6.7)^a^	0.008	0.15
	EO-d (%)	1	6.1 (1.0)^c^	5.5 (1.4)	5.1 (0.7)^a^	0.031	0.08
Follow-through	FDS-d (%)	6	6.3 (2.3) ^b^^,^^c^	4.6 (2.0)^a^	3.9 (2.4)^a^	0.020	0.08
	MD-d (%)	7	7.4 (1.5)^c^	7.1 (1.4)	6.0 (0.9)^a^	0.027	0.07
	PD-d (%)	1	26.7 (6.7)^b^	20.7 (4.6)^a^	22.5 (7.1)	0.010	0.12
	UT-d (%)	1	35.4 (4.3)^b^	32.7 (5.3)^a^	32.0 (7.1)	0.016	0.10
	UT-d (%)	3	20.3 (2.2)^c^	22.4 (2.7)	22.4 (2.3)^a^	0.029	0.07
	UT-d (%)	5	24.7 (3.3)^b^	23.0 (2.6)^a^	24.1 (3.2)	0.045	0.06
	MT-d (%)	6	41.4 (6.0)^c^	42.8 (6.5)	44.7 (4.4)^a^	0.015	0.09
	MD-b (%)	1	52.5 (6.5)^b^^,^^c^	48.8 (6.4)^a^	48.9 (4.9)^a^	0.004	0.15
	MF-b (%)	6	2.9 (0.5)^c^	2.54 (0.7)	2.52 (0.3)^a^	0.011	0.10

^a^ Significantly different from low scores, ^b^Significantly different from mid scores, ^c^Significantly different from high scores, ε²: effect size.

During the follow-through phase, the activation of the upper trapezius on the drawing side varied depending on the score for three participants. Specifically, A1 and A5 showed higher activation during low scores compared to mid scores (*p* = 0.054); while A5 exhibited lower activation during low scores compared to high scores. Differences in external oblique activation on the drawing side were observed for A3 and A6, but in different ways for each participant. For A3, activation was higher during low scores compared to mid scores. For A6, it was lower during low scores compared to high scores. The multifidus on the bow side for A6 showed greater activation during low scores compared to high scores. Moreover, the flexor digitorum superficialis also exhibited higher activation during low scores compared to both mid scores (*p* = 0.052) and high scores for A6. The medial deltoid on the drawing side was more activated during low scores compared to high scores for A7. For A1, the posterior deltoid was more activated during low scores than for mid scores. While the medial deltoid on the bow side showed more activation during low scores than during mid and high scores. For A6, the medial trapezius was less activated during low scores compared to high scores. No other differences in muscle activity were observed among the remaining muscles and participants during the follow-through phase. The muscle activity values for muscles showing significant differences for an archer during follow-through phase are presented in Table 3.

#### 3.2.7 Postural control

During the aiming phase, the mean velocity of CoP displacement was significantly higher during low scores compared to mid scores for A1 (Table 4). Conversely, for A7, CoP velocity was significantly lower during mid scores compared to high scores (*p* = 0.068). Additionally, the CoP surface area was only smaller during low scores than during the high scores. No other difference in postural control variables was observed among participants during aiming phase. The postural control values showing significant differences for individual archers during aiming phase are presented in Table 4.

**Table 4:** Postural control parameters values [median (IQR)], across scores level for each archer (S: surface, Rx and Ry: range of CoP displacement in *X* axis and *Y* axis, V, Vx, Vy and vmax: mean velocity of CoP displacement general, in *X* axis, and in *Y* axis, and maximum velocity CoP dis-placement).

Phase	Variable	Archer	Low	Mid	High	*p*-value	ε²
Aiming	S (mm²)	7	56.4 (42.4)^c^	63.9 (25.1)	75.1 (38.0)^a^	0.036	0.07
	V (mm/s)	1	20.9 (2.2)^b^	19.4 (2.2)^a^	20.0 (2.4)	0.048	0.07
	V (mm/s)	7	19.6 (1.4)	18.8 (1.7)^c^	20.1 (0.4)^b^	0.033	0.07
Follow-through	S (mm²)	1	333.0 (4.7)^b^^,^^c^	214.6 (151.9)^a^	155.2 (63.1)^a^	0.003	0.22
	S (mm²)	4	152.6 (43.9)^†^	122.6 (68.7)^a^	154.3 (60.9)	0.037	0.07
	Rx (mm)	1	8.5 (2.6)^c^	7.2 (4.7)^c^	5.0 (2.2)^a^^,^^b^	0.007	0.13
	Ry (mm)	4	24.2 (4.3)^c^	19.7 (6.7)	19.7 (5.8)^a^	0.046	0.06
	V (mm/s)	8	67.6 (11.7)^c^	63.2 (7.5)^a^	65.6 (8.2	0.041	0.08
	Vx (mm/s)	1	16.4 (2.3)^b^^,^^c^	14.6 (1.5)^a^	13.1 (2.1)^a^	0.001	0.22
	Vmax (mm/s)	1	279.8 (40.9)^b^	251.2 (29.7)^a^	243.9 (44.4)	0.224	0.09
	Vmax (mm/s)	4	293.7 (49.0)^c^	275.9 (72.6)	250.4 (58.3)^a^	0.007	0.11

^a^ Significantly different from low scores, ^b^Significantly different from mid scores, ^c^Significantly different from high scores, ε²: effect size.

During the follow-through phase, the total surface of the CoP displacement was higher for low scores compared to mid scores for A4 and compared to high scores for A1. The amplitude of CoP displacement along the mediolateral axis was greater for both low and mid scores compared to high scores for A1. Along the anteroposterior axis, a trend toward greater displacement amplitude was observed for low scores compared to high scores for A4 (*p* = 0.058). The mean CoP displacement velocity was significantly higher for low scores compared to mid scores for A8, and along the anteroposterior axis, it was higher for low scores compared to both mid and high scores for A1. The maximal velocity of CoP displacement was higher for low scores compared to mid scores for A1 and compared to high scores for A4. No other difference was observed among archers during the follow-through phase. The postural control values showing significant differences for an archer during follow-through phase are presented in Table 4.

#### 3.2.8 Body configuration

No significant differences were observed in kinematic variables among participants during both aiming and follow-through phases.

#### 3.2.9 Temporal strategies

As shown in Table 5, aiming duration was significantly shorter during low scores compared to high scores for A8, and significantly longer during low scores compared to mid scores for A1. No significant differences in MCRT were observed across participants, nor in aiming duration for other archers.

**Table 5:** Duration of aiming [median (IQR)], across scores level for each archer (aiming time: duration of aiming).

Phase	Variable	Archer	Low	Mid	High	*p*-value	ε²
Aiming	Aiming duration	1	2.2 (0.7)^b^	1.6 (0.71)^a^	2.1 (0.7)	0.023	0.09
	Aiming duration	8	2.0 (0.8)^c^	2.5 (0.8)	3.7 (1.2)^a^	0.007	0.15

^a^ Significantly different from low scores, ^b^Significantly different from mid scores, ^c^Significantly different from high scores, ε²: effect size.

## 4 Discussion

This study aimed to investigate the primary determinants of performance in high-level archery by examining postural control, neuromuscular activation, body configuration, and temporal strategies to differentiate between factors that are consistent across a group of eight experienced archers and those that reflect individual specific adaptations, particularly during the aiming and follow-through phases.

At the group level, several parameters demonstrated a significant association with performance. During the aiming phase, these included the activity of seven muscles, three postural control parameters, and the MCRT. In the follow-through phase, four muscles and three postural control parameters were also identified as significant determinants.

At the individual level, an analysis of the aiming phase highlighted distinct, athlete-specific performance predictors. Regarding neuromuscular activation, the middle deltoid of the bow-side arm was a significant factor for three athletes. In contrast, three distinct drawing-side muscles were each significant for only a single archer. The surface of the CoP was associated with performance for one archer, and global velocity of CoP was significant for two archers. Aiming duration differed between two archers, but with opposite trends. This inter-individual variability persisted into the follow-through phase. Within the neuromuscular domain, while the upper trapezius and external oblique of the drawing side were significant determinants for three and two archers respectively, six other muscles were each significant for only a single athlete. Four postural control parameters were significant for one archer each, while maximal velocity and surface of CoP displacement were associated with performance for two archers.

### 4.1 Collective determinants of performance

#### 4.1.1 Neuromuscular activity

During the aiming phase, previous studies have reported that higher activation of posterior deltoid and middle trapezius on the draw side is linked to better performance when comparing novice and elite archers (12, 13). Conversely, the current investigation of elite-level archers identified an opposite trend: lower activation in these muscles was linked to better scores. The observed discrepancy may be due to the superior performance level of the present cohort. While the participants in the Simsek et al. (12) study scored approximately 575/720, the archers in this study averaged a score of 655/720, suggesting they represent a higher tier of elite performance.

This suggests that the best archers use their muscles more efficiently. Specifically, they may rely more on small, deep shoulder muscles for stability rather than on larger, energy-demanding muscles. This efficiency reduces unnecessary muscle contractions. This allows them to reduce the tremors that can make their aim less steady. Ultimately, this better control gives them a more stable and less tiring posture, contributing to better scores.

Unlike earlier studies that linked greater draw-side extensor digitorum and flexor digitorum superficialis activation to lower performance (8, 12–14), this study found a positive association between the activation of these muscles and shooting performance. The higher proficiency level of the participants in the present study, compared to those in the investigation by Simsek et al. (12), suggests that highly experienced archers may adopt different muscle recruitment strategies, with more refined and consistent neuromuscular control (33). These differences may also be attributable to methodological variations between studies, including differences in instrumentation and experimental protocols.

During the aiming and follow-through phases, previous literature has indicated that on the bow side, increased activation of the flexor digitorum superficialis and decreased activation of the extensor digitorum are associated with inferior performance outcomes (18). In contrast, the present study identified a divergent trend. Flexor digitorum superficialis activation was positively associated with performance, while extensor digitorum superficialis activation was negatively.

Figures 2b,3a show the difference in muscle activity between low and high-scoring shots which is primarily attributable to sporadic instances of high-amplitude activation occurring during low-score shots. Such activation peaks are absent in high-scoring shots. This suggest that moderate extensor digitorum activation supports high performance, whereas excessive activation may impair accuracy. Similarly, an optimal level of flexor digitorum superficialis activation may be crucial for maintaining stability and control, whereas insufficient activation could impair performance. These interpretations should be approached with caution. The comparative analysis is based on a singular study from the literature. These presents findings do not fully match its reported patterns. The precise contribution of these muscles to elite archery performance remains unclear, representing a clear avenue for future investigation.

Among the core muscles assessed during the aiming phase, higher multifidus activation on the draw side was associated with better performance. This align with Azhar et al. (16). However, our study extends their work by providing a bilateral assessment of multifidus activation, a methodological aspect not included in their original investigation. The multifidus plays a key role in maintaining lumbar stability and facilitating force transfer between the trunk and upper limbs (34). That only the draw-side multifidus showed heightened activation in high-scoring shots suggests that asymmetric trunk stability is a key determinant of performance in archery. This increase may improve control of trunk rotation, enhanced postural stability, and boost shooting precision.

During the follow-through phase, activation of the draw-side external oblique was higher for high-scoring shots compared to low-scoring ones. This finding appears to be novel. To our knowledge no previous studies have specifically examined the role of the external oblique during the follow-through phase in archery. Increased external oblique on draw-side side activation may be functionally important for controlling the rotational torques of the asymmetric upper limbs while simultaneously maintaining the axial alignment of the trunk. Enhanced control of core muscles could mitigate postural disturbances (35, 36), improve shot-to-shot consistency, and contribute to a cleaner and more stable release. A notable feature of high-scoring shots in the elite archers examined was reduced transvs. abdominis activation during the follow-through phase. This diverges from Azhar et al. (16), who reported higher activation in elite archers compared to novices.

However, this study analyzed the entire follow-through phase (16),whereas the present analysis focused on the 500 ms immediately after the arrow release. Additionally, Azhar et al. (16) compared different skill-level groups (elite vs. novice), while this analysis examined intragroup performance differences among elite archers. These methodological differences may explain the discrepancies. The lower transvs. abdominis activation observed in this study may reflect a more efficient motor strategy in elite archers, requiring less core engagement immediately after release.

#### 4.1.2 Postural control

During the aiming phase, several CoP velocity metrics were high in low-performance shots. Specifically, this was applied to global CoP velocity, as well as its components along the anteroposterior and mediolateral axes. These results indicate that enhanced stability of the CoP during the aiming phase is a key determinant of high-level performance. Such findings are not unique to archery and have been reported in other precision sports. For example, superior performance in rifle shooting is associated with lower CoP velocity (21, 37), and greater shooting accuracy in biathlon is linked to minimal body sway (38, 39).

In the context of archery, diminished velocity of CoP displacement during aiming is a key determinant of high-scoring shots (6, 14, 40). Moreover, mediolateral CoP displacement was correlated with bow movement in both vertical and horizontal directions (6), suggesting that controlling CoP displacement may improve bow stability during aiming. Overall, these findings suggest that CoP velocity is not only a distinguishing factor between elite and novice archers but also a key determinant of performance within elite-level archery, in all movement directions.

During follow-through phase, greater CoP range in the anteroposterior axis and higher maximum velocity were observed in low scoring shots. These results are consistent with findings from a previous study on 31 elite archers (25). However, one group-level result differed with mediolateral CoP range was higher in high-scoring shots. This unexpected finding may suggest that controlled movement in mediolateral direction contributes positively to optimized release mechanics. Rather than indicating instability, this pattern could represent a strategic and functional adjustment. This aims to facilitate a more biomechanically efficient and accurate release, a characteristic less evident in low-scoring shots.

#### 4.1.3 Temporal parameters

Shorter MCRT were associated with higher scores, confirming previous results by Ertan et al. (11) and Spratford and Campbell (25). These results suggest that elite archers improve performance by reducing MCRT and controlling CoP movements during follow-through.

In summary, these findings indicate that elite archers often displayed opposite muscle activation patterns. Lower activation in specific arm muscles may signify more efficient draw and hold strategies. In contrast, enhanced activation of core muscles such as the multifidus and external oblique was essential for trunk stability and control. Better postural stability, indicated by slower CoP movements, and shorter MCRT, were also associated with higher scores.

Parameters identified as group-level strategies were considered representative of the entire group and were therefore excluded from individual-level analysis. Where a parameter was significant at both levels, the group-level finding was prioritized in interpretation. This approach assumes that individual strategies are part of the group's overall strategic behavior. Including both levels in the analysis could create redundancy, since the group-level strategy already reflects the main trends shared by individuals within the group.

### 4.2 Individual determinants of performance

#### 4.2.1 Neuromuscular activity

In the aiming phase, distinct inter-individual differences emerged in muscle activation patterns. On the drawing side, one archer exhibited higher middle deltoid activation during lower performance trials, despite prior research (12, 13) generally associating increased activation of this muscle with better performance, suggesting that this archer may employ a unique strategy.

For three archers, lower activation of the bow-side middle deltoid was linked to better performance. This suggests that deliberately reducing activity in this key shoulder muscle, which typically provides crucial support for the bow arm (19), may be an individualized strategy to enhance stability and precision. Prior to release, one archer showed higher external oblique activation on the drawing side during lower-performance trials. Although this observation aligns with Matsunaga, Imai, and Kaneoka (20), it likely reflects a compensatory mechanism rather than a common motor pattern among elite archers.

During the follow-through phase, higher-scoring shots were characterized by reduced activation of the drawing arm muscles (FDS, MD, PD). These finding contrasts with previous studies (11, 14, 17) and suggests that minimizing post-release muscle activity may benefit some archers depending on their technique. These findings also highlight previously underexplored roles of the upper and middle trapezius on the draw side, and the middle deltoid on the bow side, during the follow-through phase. In contrast, one archer exhibited lower activation of the bow side multifidus during lower-scoring shots. This result diverges from Azhar et al. (16) and may indicate a deficit in postural control or trunk stability during less successful attempts.

#### 4.2.2 Postural control

During the aiming phase, one archer exhibited a larger CoP surface area during higher-scoring shots, which is contrary to trends in other precision sports, where reduced sway typically enhances stability and precision (21–24). This may indicate an individualized strategy in which subtle postural adjustments optimize visual alignment.

During the follow-through phase, despite individual differences in metrics, increased postural sway was associated with lower scores. Specifically, lower scores were linked to higher global velocity for one archer, with anteroposterior velocity for another, and with CoP surface area for two others. These findings, align with previous research showing that minimized postural sway benefits performance, suggest that refined, individualized postural strategies to limit unnecessary movement post-release may reflect advanced motor control and greater experience.

#### 4.2.3 Temporal parameters

Previous literature have shown that shorter aiming durations are associated with superior performance (6, 8, 14). While the present study identified aiming duration as a significant factor for two archers, the effect was inconsistent. One archer's performance improved with shorter durations, while the other's improved with longer durations. This variability reflects personal techniques or habits and underscores the importance of incorporating individual-level analysis when evaluating performance outcomes. In fact, some archers may benefit from quicker aiming to mitigate physical fatigue and minimize excessive cognitive processing (41), while others need more time to reach optimal focus. These findings highlight the need to tailor aiming strategies to individual needs, especially at the elite level.

These results suggest that elite archers develop highly individualized strategies to improve their performance. These include selective muscle activation (e.g., middle deltoid during aiming, upper trapezius during follow through), individualized postural adjustments to reduce movement after release, and customized aiming durations. Recognizing these individual differences reinforces the importance of personalized training approaches based on each archer's unique biomechanical and neuromuscular profile.

### 4.3 Practical applications

At the group level, the activation patterns of four muscles in the drawing arm during the aiming phase demonstrated a significant association with performance. However, these findings contrast with previous studies comparing elite and novice archers. This discrepancy suggests that while certain activation patterns may facilitate progression to elite level, further refinements in muscle activation strategy could distinguish top performers. Optimal control of the bow side appears to involve a dual neuromuscular requirement. Maintaining a well-regulated level of extensor digitorum activity to prevent excessive tension, while simultaneously ensuring sufficient flexor digitorum superficialis activation to preserve grip stability. For coaches, this highlights the need to help athletes achieve optimal bow-arm muscle activation to improve performance.

Reduced CoP velocity during the aiming phase is a key determinant of high-accuracy shots. Coaches could integrate balance training, core strengthening, and proprioceptive exercises into training regimens to specifically enhance postural stability. Conversely, greater anteroposterior CoP displacement and peak velocity during follow-through were associated with decreased scores, indicating that post shot instability can negatively impact performance. Interestingly, a larger mediolateral CoP range was associated with higher scores, indicating that controlled movement can help absorb release forces and maintain postural alignment. Based on these findings, training programs could be designed to minimize anteroposterior CoP displacement and velocity during the follow-through, while preserving a degree of functional mediolateral sway.

MCRT also emerged as a key performance determinant, consistent with previous research (25). A shorter reaction time following the clicker was linked to better scores. To improve this, training interventions could be designed to enhance the speed and precision of the motor response following the auditory cue of the clicker. Targeted drills focused on enhancing temporal consistency, attentional control, and the fluidity of the follow-through may contribute to a reduction in reaction time and an increase in overall shot efficiency.

At the individual level, some archers demonstrated patterns of reduced muscle activation, such as lower middle deltoid activity on the bow side during aiming and diminished upper trapezius activity on the drawing side during the follow-through. Their inconsistency across all archers suggests that the development of individualized motor strategies is a characteristic determinant, even at the elite level. Therefore, coaches should evaluate and accommodate each archer's specific muscle activation patterns, rather than applying a uniform training model.

In the follow-through phase, some archers may have adopted unique strategies to enhance postural control. Evaluating impact of post-shot balance on performance should be individualized, as not all archers appeared to benefit equally. Similarly, the relationship between aiming duration and performance varied across archers, further supporting the need for personalized coaching approaches. Therefore, tailoring aiming duration to an athlete's individual characteristics may be a viable strategy for enhancing shot consistency and accuracy.

### 4.4 Limitations

This study provides valuable insights into the biomechanics of elite archery. However, one limitation must be acknowledged. All participating archers were trained by the same coach, which may have led to shared technical habits. These techniques might differ from those used by other elite archers trained under alternative coaching strategies, thus limiting the generalizability of the findings. Indeed, the technical framework provided by this coach (e.g., imposing a particular technique on the entire group) may also prevent us from identifying individual strategies that would be present in a more diverse group of archers.

However, this limitation should be contextualized. Prior to joining their current coach, the athletes had extensive training histories under various coaching philosophies. Therefore, their fundamental shooting techniques must be already established.

Furthermore, we acknowledge a minor limitation regarding instrumentation. Specifically, while the IMU system has been previously validated, this study did not include direct validation for the EMG and force platform systems, though references supporting their use in similar conditions were provided. Given this study emphasis on high-precision measurements, we recommend that future research in elite sports biomechanics prioritizes device-specific validation to guarantee the highest level of data accuracy.

Another limitation is the absence of fatigue assessment during the 72-arrow shooting session. Given that muscular fatigue affects both EMG (42) and postural control (43), its influence on performance cannot be overlooked.

Finally, a small group of only 8 archers was involved, so the results must be interpreted with caution. In short, this study offers some interesting ideas, but more research is needed with many more archers from different backgrounds to confirm that these findings apply to all elite athletes.

### 4.5 Perspectives

During the follow-through, superior performance was associated with a distinct neuromuscular pattern: higher activation of the flexor digitorum superficialis and lower activation of the extensor digitorum, these finding contrasts with previous research. These discrepancies highlight the need for further investigation into the neuromuscular mechanisms underlying performance.

Additionally, this study also identified potential relationships between shooting performance and two other variables: the behavior of the bow arm muscles during the follow-through and the activation patterns of core muscles. These domains remain largely unexplored in archery research. The relationship between core muscles and postural control appears to be significant for performance. This suggests that further investigation in this domain could offer deeper insights into how archers manage their posture and stability. Exploring these overlooked aspects may yield a more comprehensive understanding of the biomechanical strategies that contribute to elite performance in archery.

In addition, quantifying fatigue would enable the investigation of its potential influence on the observed performance parameters, thereby establishing a clearer link between the athlete's physiological state and the measured data.

Finally, a statistical approach that defines the weight of each factor could significantly advance archery research and provide additional insights to coaches, helping them better understand how to optimize performance.

## 5 Conclusions

This study identifies key individual strategies across multiple performance domains at the elite level: neuromuscular (e.g., the reduction of bow-side middle deltoid and draw-side upper trapezius activity), postural (e.g., the control of CoP surface area during follow-through), and temporal (e.g., the adaptation of aiming duration). At the group level, results on muscle activation in the aiming phase appear inconsistent with previous research, suggesting a need for further investigation. Furthermore, the specific functional roles of the forearm during the follow-through and core muscles warrant more detailed investigation. Reduced center of pressure velocity during aiming, as well as decreased anteroposterior range and peak velocity in the follow-through phase, were associated with improved performance. Lastly, clicker reaction time was confirmed as a key factor for higher scores.

In summary, training programs should first focus on establishing a solid technical foundation (stability, muscular balance when holding the bow, reaction to the clicker), followed by a phase of personalization where these skills are adapted based on careful observation of the archer's individual motor strategy. Finally, future research should aim to quantify the relative contribution of these variables to refine elite training protocols and improve performance assessment in archery.

## Data Availability

The raw data supporting the conclusions of this article will be made available by the authors, without undue reservation.
